# Development and Application of a Label-Free Fluorescence Method for Determining the Composition of Gold Nanoparticle–Protein Conjugates

**DOI:** 10.3390/ijms16010907

**Published:** 2014-12-31

**Authors:** Dmitriy V. Sotnikov, Anatoly V. Zherdev, Boris B. Dzantiev

**Affiliations:** A.N. Bach Institute of Biochemistry, Russian Academy of Sciences, Leninsky Prospect 33, Moscow 119071, Russia; E-Mails: sotnikov-d-i@mail.ru (D.V.S.); zherdev@inbi.ras.ru (A.V.Z.)

**Keywords:** gold nanoparticles, protein conjugates, tryptophan fluorescence

## Abstract

A method was developed for determining the composition of the conjugates between gold nanoparticles and proteins based on the intrinsic fluorescence of unbound protein molecules. The fluorescence was evaluated after separation of the conjugates from the reaction mixture by centrifugation. Gold nanoparticles obtained using the citrate technique (average diameter 24 nm) were conjugated at pH 5.4 with the following four proteins: human immunoglobulin G (IgG), bovine serum albumin (BSA), recombinant streptococcal protein G (protein G), and Kunitz-type soybean trypsin inhibitor (STI). The compositions of these conjugates were determined using the developed method. The conjugate compositions were dependent on the concentration of the added protein, and in all cases reached saturation. The equilibrium dissociation constants of the gold nanoparticle conjugates with IgG, BSA, protein G, STI in the initial section of the concentration dependence curve were 4, 6, 10, and 15 nM, respectively. Close to saturation, the corresponding values were 25, 76, 175, and 100 nM, respectively. The maximal binding capacities of a single gold nanoparticle for IgG, BSA, Protein G, and STI were 52, 90, 500, and 550, respectively, which agrees well with the hypothesis of monolayer immobilization.

## 1. Introduction

Gold nanoparticles are widely used in various fields of biochemistry, medicine, and analytical chemistry because of their unique physical and chemical properties [1,2,3,4,5,6]. In most cases they are modified with proteins or nucleic acids before application [3,7,8]. The preparation of such complexes has resulted in high demand for the development and improvement of methods to characterize their composition and functional properties.

Despite the intense development of this field of knowledge in recent decades, descriptions of the interactions between proteins and gold nanoparticles remain controversial. Some studies [9,10,11] state that proteins form a monolayer on the surface of gold nanoparticles, whereas other studies [12,13] describe a multilayer immobilization. De Roe *et al*. [14] found that protein A interacted with gold nanoparticles with an equilibrium dissociation constant of 343.9 nM. By contrast, Ghitescu *et al*. [15] found that the dissociation constant at low concentrations of protein A was 2.3 nM, and at high concentrations was 500–900 nM. Both these studies were performed using X-ray spectroscopy. Large differences (up to five orders of magnitude) among the dissociation constants have also been observed for gold nanoparticles with other proteins, such as bovine serum albumin (BSA) [11,14,16,17,18]. The question of cooperativity of the sorption of proteins on nanoparticles also remains unanswered. Data obtained by Lacerda *et al*. [12] demonstrate that the process of sorption of serum proteins on gold nanoparticles can be characterized by both positive and negative cooperativity depending on the size of the nanoparticles. For example, insulin demonstrates a positive cooperativity for sorption on gold particles with a diameter of 100 nm (Hill coefficient 3.64), and negative cooperativity for particles with a diameter of 20 nm (Hill coefficient 0.63).

Because of these uncertainties, a detailed characterization of colloidal conjugates with proteins is required. There are many analytical methods currently available for determining the composition of these conjugates [19]. To obtain accurate information, label-free is preferred because of labeling of any molecule leads to changes in its properties. A label-free method also reduces the number of sample preparation steps, which is desirable as additional steps increase the error in the final value. Tryptophan fluorescence is a promising method for studying the interaction of proteins with nanoparticles because it is present in almost all proteins. This method has been exclusively applied as detection of tryptophan fluorescence caused by the interaction of proteins with nanoparticles [12,18,19,20,21,22]. However, it is known that quenching of the fluorophore by nanoparticles, including gold nanoparticles, is a complex multifactorial process with non-linear dependencies, and in some cases enhancement of the fluorescence is observed instead of quenching [23]. This could have caused the large differences observed among the dissociation constants (range 0.88·10^−4^ M [21] to 1·10^−9^ M [18]) for BSA-gold nanoparticles complexes determined by this method.

In our work, we used an alternative approach to determine the composition of the gold nanoparticle conjugates with various proteins. This method was based on measurement of the residual fluorescence of the reaction solution after separation from nanoparticles by centrifugation. This approach eliminates the influence of nanoparticles on the protein fluorescence, and allows determining the amount of unbound protein with high accuracy. The following four proteins were selected for this study: human immunoglobulin G (IgG); bovine serum albumin (BSA); a recombinant mutant of protein G from *Streptococcus* spp. (protein G) formed from three IgG-binding fragments [24]; and Kunitz-type soybean trypsin inhibitor (STI). These proteins were chosen because of their frequent use in immunochemical systems as carrier proteins for low molecular weight haptens (BSA and STI), as receptor molecules (IgG and protein G), and as nanoparticle stabilizers and components of the reaction medium (BSA). In addition, serum albumin and IgG are major protein components in blood [25]. If nanoparticles enter the bloodstream they will mainly interact with these proteins [12,26]. Therefore, the study of these proteins is of great importance for medicine and toxicology.

The aim of this study was to develop and test a new label-free method for the characterization of gold nanoparticle-protein conjugates. Potentially, this method could be used also to characterize protein conjugates of other nanoparticles.

## 2. Results and Discussion

### 2.1. Measurement of the Dimensions of the Gold Nanoparticles

**Figure 1:**
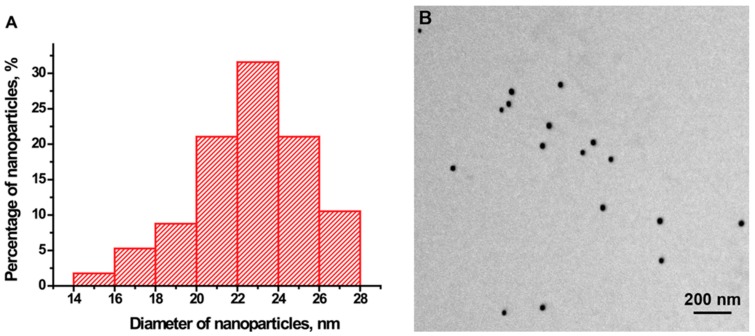
Characteristics of the gold nanoparticles. (**A**) Histogram of particle size distribution; (**B**) An image of gold nanoparticles from the electron micrograph.

Electron microscopy of the gold nanoparticles showed a high degree of size uniformity. The 57 tested particles had an average diameter of 23.9 ± 2.7 nm, and an axial ratio of 1.20 ± 0.09 (Figure 1). The hydrodynamic diameter of the particles according to data from dynamic light scattering was 25.8 ± 1.5 nm.

### 2.2. Using the Protein’s Intrinsic Fluorescence to Determine the Composition of the Protein-Gold Nanoparticle Conjugates

The vast majority of proteins have the intrinsic fluorescence, which occurs mainly because of their tryptophan content. Tryptophan has a maximum absorption at 280 nm and emission maximum at 340–360 nm [27].

The proposed method for determining the composition of the protein-gold nanoparticle conjugates is based on comparison of the initial fluorescence of calibration solutions (F_0_) and the residual fluorescence of the reaction solutions after separation of the synthesized conjugates (F). The difference between these two values corresponds to the amount of the protein in the conjugate as shown by the following equation:

(C_0_ − C_unbound_)/C_0_ = (F_0_ − F)/F_0_ C_conjugated_ = C_0_ − C_unbound_ = C_0_ (F_0_ − F)/F_0_ (1)

where C_0_ is the initially added protein concentration, C_unbound_ is the protein concentration in the reaction solutions after separation of the synthesized conjugates, and C_conjugated_ = is the decrease of protein concentration reflecting its binding with the nanoparticles in the course of the conjugation.

**Figure 2:**
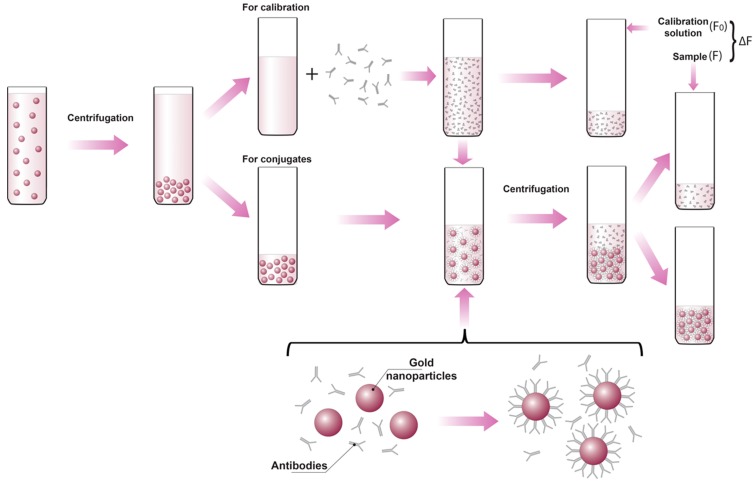
Experimental protocol for determining the composition of gold nanoparticle-protein conjugates using immunoglobulin G (IgG) as an example. F_0_ is the fluorescence of the calibration solutions, F is the fluorescence of the supernatant after centrifugation of the synthesized conjugates, and ∆F is given by F_0_ − F.

The experimental setup shown in Figure 2 allows measurement of the fluorescence of proteins in calibration solutions and in supernatants under the same conditions.

The fluorescence of tryptophan is dependent on a number of factors, including the composition of its immediate environment. Even small changes in the ionic composition of the solution and in the conformation of the protein can significantly affect the fluorescence signal [27,28,29]. We have compared BSA fluorescence in distilled water, in citrate solution used for the synthesis of gold nanoparticles in the supernatant after centrifugation of the nanoparticles. The three obtained dependences of the BSA fluorescence at 350 nm from its concentration have different slopes of their linear approximations: (1) BSA fluorescence in water −18.4 arb. units of fluorescence per 1 μg/mL of BSA in the solution; (2) BSA fluorescence in citrate −20.4 arb. units per 1 μg/mL; (3) BSA fluorescence in supernatant −19.8 arb. units per 1 μg/mL (accuracy of measurements ≤ 1%).

So, to use guaranteed proper control for comparison and eliminate all potential risks caused by influencing compounds, we recommend comparing the fluorescence of non-adsorbed protein with supernatant solution. Thus, the calibration solutions were prepared using the supernatant (pH 5.4) obtained after centrifugation of gold nanoparticles. The calibration and test samples were measured simultaneously using the same microplate (See Supporting Information 1). Furthermore, because the microplates adsorb protein, which can affect the fluorescence, the measurements were performed immediately after transferring the samples into wells of the microplate.

### 2.3. Determination of the Concentration of the Gold Nanoparticles

The electron microscopy data showed the obtained particles had an average diameter of 24 nm, which was used to obtain a particle volume of v = 7.2 × 10^−24^ m^3^. Taking into consideration the density of gold (19,300 kg/m^3^), we calculated the mass of one particle as 1.4 × 10^−16^ g. For the synthesis, 0.2 mL of 5% HAuCl_4_ was added to 100 mL of water. Therefore, the concentration of gold in a solution was 0.05 g/L, and, the number of particles per milliliter was *n* = 3.6 × 10^11^.

### 2.4. Determination of the Amount of Protein Adsorbed on the Gold Nanoparticles

We have used values of the fluorescence at 350 nm as well as it corresponds to the highest peak of the spectra and also is in accordance to common practice of the characterization of protein fluorescence [27,28,29]. Individual spectral properties of each system didn’t influence on linearity of the studied main fluorescence at 350 nm as well as on calculation of proteins content in the studied solutions.

**Figure 3:**
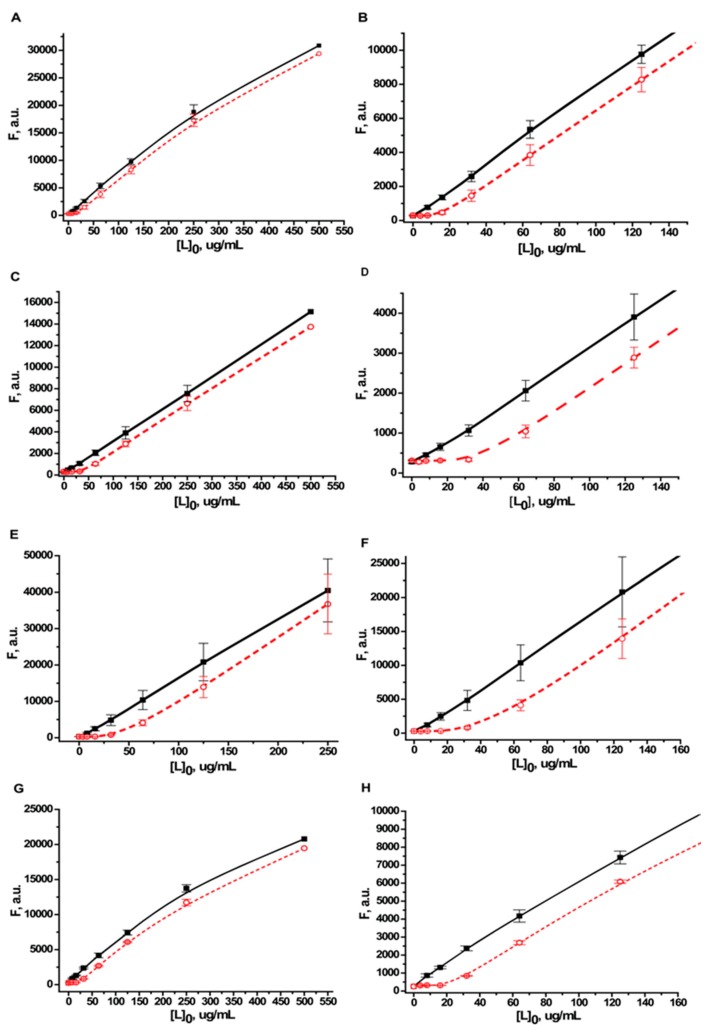
The dependence of the fluorescence intensity of proteins on their concentrations. Fluorescence intensity (F) at 350 nm in the calibration solutions [L]_0_ (solid squares) and in the supernatants obtained after centrifugation of the conjugates with gold nanoparticles (open circles). Graphs (**A**,**B**) are for bovine serum albumin (BSA) (average of six repetitions); (**C**,**D**) are for Kunitz-type soybean trypsin inhibitor (STI) (average of three repetitions); (**E**,**F**) are for recombinant streptococcal protein G (protein G) (average of three repetitions); and (**G**,**H**) are for human immunoglobulin G (IgG) (average of three repetitions). Graphs **B**, **D**, **F**, **H** show the initial parts of graphs **A**, **C**, **E**, **G**.

The dependency of the fluorescence intensity at 350 nm on the concentration of added protein was determined in the calibration solutions and in the test samples (Figure 3). In the concentration range of 0–250 μg/mL, dependence of the intensity of the protein fluorescence on the concentration in the calibration solution was linear (*R* was >0.99 for all proteins). However, as the fluorescence increased, so did the absolute error in the measurements. Consequently, only the data obtained for proteins with concentrations ≤125 μg/mL were used to calculate the conjugates composition. At higher concentrations, the measurement error was greater than the difference between the values of the fluorescence for the calibration solutions and for the tested samples.

At low protein concentrations in the synthesis (≤8 μg/mL), the quantity of unbound molecules of protein was below the detection limit of the method (0.5 μg/mL). In this case, virtually all the added protein was adsorbed on the surface of the nanoparticles (Figure 3). As the concentration of added protein increased, the curves for the calibration solutions and samples became parallel, which indicates saturation was reached.

Based on the difference between the signals for the samples and calibration solutions and on the quantity of gold nanoparticles in the solution, which was 1.8 × 10^12^ particles/mL taking into consideration the five-fold increase in the concentration after centrifugation of the conjugate and redissolution of the pellet, the quantity of immobilized protein molecules per particle was calculated according to the following equation:

[(F_0_ − F)/F_0_] × ([L_0_]/[R_0_]) = RL (2)

where F_0_ is the fluorescence of the calibration solution, F is the fluorescence of the supernatant obtained after centrifugation of the conjugate, [L_0_] is the concentration of the added protein, [R_0_] is the concentration of nanoparticles, and RL is the quantity of protein molecules bound to a single gold nanoparticle.

**Figure 4:**
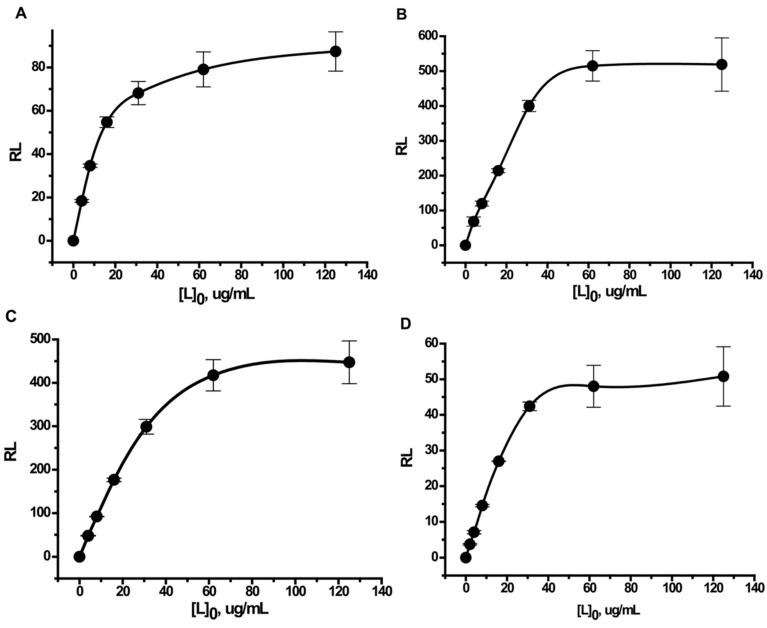
Number of molecules of protein adsorbed on a single nanoparticle. The dependence of the number of molecules of protein adsorbed on a single nanoparticle (RL) on the protein concentration used in the synthesis ([L_0_]). Graph (**A**) is for bovine serum albumin (BSA); (**B**) is for Kunitz-type soybean trypsin inhibitor (STI); (**C**) is for recombinant streptococcal protein G (protein G); and (**D**) is for human immunoglobulin G (IgG).

The obtained values of protein adsorbed on a single nanoparticle for different added concentrations of four studied proteins are given as curves at Figure 4. As can be seen, the saturation of adsorption capacity is reached for all cases. The concentrations necessary for the saturation lie in the range from 40 to 60 μg/mL.

### 2.5. Determination of the Equilibrium Dissociation Constants of the Protein-Nanoparticle Interaction and the Number of Sorption Sites

The constants of the protein-nanoparticle interaction were determined by the Scatchard method [30] in accordance with the following equation:

RL/[L] = (N − RL)/K_D_ (3)

where [L] is the concentration of free protein, RL is the amount of protein molecules bound to a single gold nanoparticle, K_D_ is the equilibrium dissociation constant of the complex, and N is number of binding sites on a single gold nanoparticle.

We determined the K_D_ values from curves of the dependence of the RL/[L] ratio on RL. The K_D_ was equivalent to the cotangent of the angle of the tangent. The K_D_ and N were calculated for the protein concentrations that had the following characteristics: (i) a large difference between the supernatant and background signals; and (ii) did not reach saturation. According to (i), protein concentrations ≥16 μg/mL were selected; and for (ii) the range was <125 μg/mL. The point of the intersection between the horizontal axis and the linear section of the graph at protein concentrations close to saturation gives the total number of sorption sites on a single nanoparticle.

**Figure 5:**
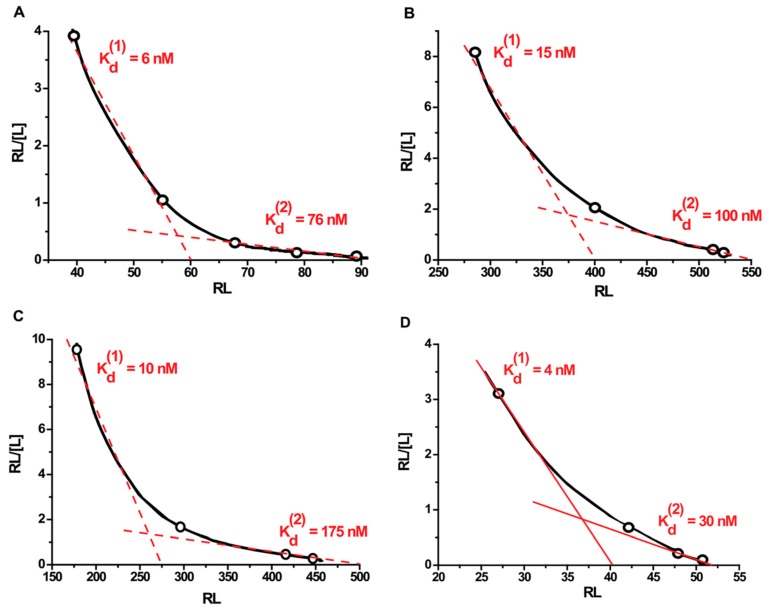
Scatchard plots of binding of proteins with gold nanoparticles. Graph (**А**) is for bovine serum albumin (BSA); (**B**) is for Kunitz-type soybean trypsin inhibitor (STI); (**C**) is for recombinant streptococcal protein G (protein G); and (**D**) is for human immunoglobulin G (IgG). K_d_^(1)^ is the equilibrium dissociation constant for conjugates at protein concentrations below the saturation point, K_d_^(2)^ is the equilibrium dissociation constant for conjugates at a protein concentration close to the saturation point. [L] is the concentration of free protein, and RL is the number of protein molecules bound to a single gold nanoparticle.

Plotting the data in Scatchard coordinates produced hyperbolic curves (Figure 5). The slope decreased with increasing protein concentration, which indicates the average strength of the association of proteins to the gold surface decreased. This is indicative of an anti-cooperative mechanism of the interaction.

For quantitative characterization of the strength of the binding, we calculated the average values of the equilibrium dissociation constants for the following two linear sections of the Scatchard graph: concentrations significantly below the saturation value (K_d_^(1)^), and concentrations close to the saturation value (K_d_^(2)^). The point of intersection of the linear finite section of the chart with the *x*-axis gives the maximum number of binding sites (N). All data obtained are summarized in Table 1.

**Table 1:** Parameters for protein adsorption on gold nanoparticles.

Protein	MW, kD	1/MW, kD^−1^	K_d_^(1)^, nM	K_d_^(2)^, nM	*N*
IgG	150	0.00667	4	30	52
BSA	66	0.01515	6	76	90
Protein G	26	0.03846	10	175	500
STI	20	0.05	15	100	550

K_d_^(1)^ and K_d_^(2)^ are the equilibrium dissociation constants for sections of the Scatchard graph significantly below the saturation value, and close to the saturation value, respectively; *N* is the maximum number of binding sites; BSA: bovine serum albumin; STI: Kunitz-type soybean trypsin inhibitor; protein G: recombinant streptococcal protein G; IgG: human immunoglobulin G.

The values of the obtained dissociation constants generally increased with increasing molecular weight of the proteins. This trend is consistent with earlier data. Thus, De Roe *et al.* [14] interpreted their data about increase in affinity for larger proteins as a result of multiple contacts, but allowed involvement of other factors. The further studies confirmed significant impact of hydrophobic and electrostatic forces in the formation of the nanoparticle-protein complexes (see review [31]). However, individual properties of adsorbed proteins influence significantly on the role of different binding forces. For example, Zhang *et al.* [32] recently studied different serum proteins and indicated five mechanisms of their adsorption.

**Figure 6:**
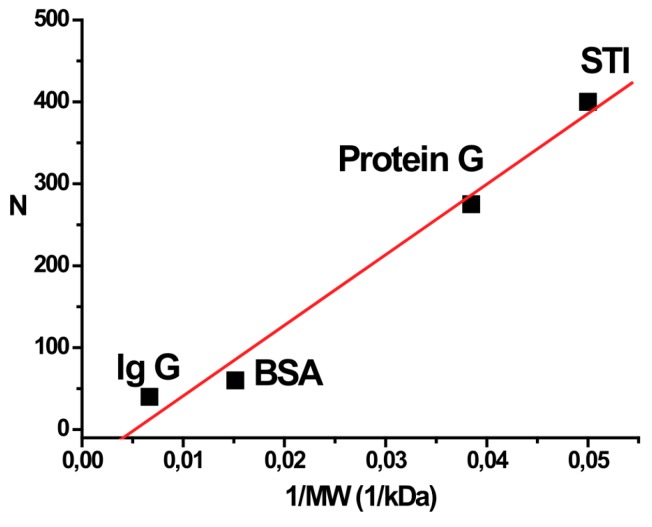
Correlation between binding sites and molecular weight of the protein. Dependence of the number of binding sites (N) on a single gold nanoparticle on the reciprocal value of molecular weight of the protein. BSA: bovine serum albumin; STI: Kunitz-type soybean trypsin inhibitor; protein G: recombinant streptococcal protein G; IgG: human immunoglobulin G.

The decreasing slope of the Scatchard curves with increasing protein concentration may be explained by steric hindrance between adjacent adsorbed protein molecules when their density on the surface of the particle increases. The number of sites for saturation sorption (N) has an almost linear dependence on the reciprocal value of the protein’s molecular weight (Figure 6). These data also accord to studies of De Roe *et al*. [14] with good correlation of available surface for binding and protein/nanoparticle ratio in the complexes formed under saturated conditions. However, different factors, such as shape, amino acid composition, distribution of hydrophilic/hydrophobic regions, also influence adsorption of proteins. Such individual variations are presented, for example, in the work of Lacerda *et al*. [12].

The consideration of proteins adsorption on the nanoparticles surface should include the analysis of possible structural changes of proteins after immobilization and corresponding changes in their shape and interacting surface. The literature data contain different examples of changes in protein structure after adsorption as well as absences of registered changes [33]. Recent model of the adsorption process [34] states three-step interaction including an initial reversible association step, a rearrangement/reorientation step on the nanoparticle surface, and a final cysteine-dependent “hardening” step. However, the degree of structural changes varies significantly and depends on individual properties of studied proteins as well as from their surface density on the nanoparticle carrier in the course of adsorption (see [35] as example). In any case, our correlations of binding sites and affinity with molecular weight did not violated largely by individual characteristics of proteins potentially related to their denaturation-caused rearrangements.

Results of some earlier studies show that proteins can be adsorbed on the surface of gold nanoparticles forming several layers [12,13,36], while other researchers have argued that the proteins form a monolayer on the surface of nanoparticles [9,10,11]. This difference in the number of layers may arise from changes in the conditions of the conjugate synthesis (pH, ionic strength, composition of the reaction mixture, particle size, *etc*.). In the course of the adsorption proteins generally retain their size. Thus, correlation between the thickness of monolayer protein corona and linear size of adsorbed proteins was demonstrated in [37]. Our data for the numbers of adsorbed proteins and their comparison with the size of the protein molecules suggest that monolayer immobilization takes place for all the proteins under our conditions.

**Figure 7:**
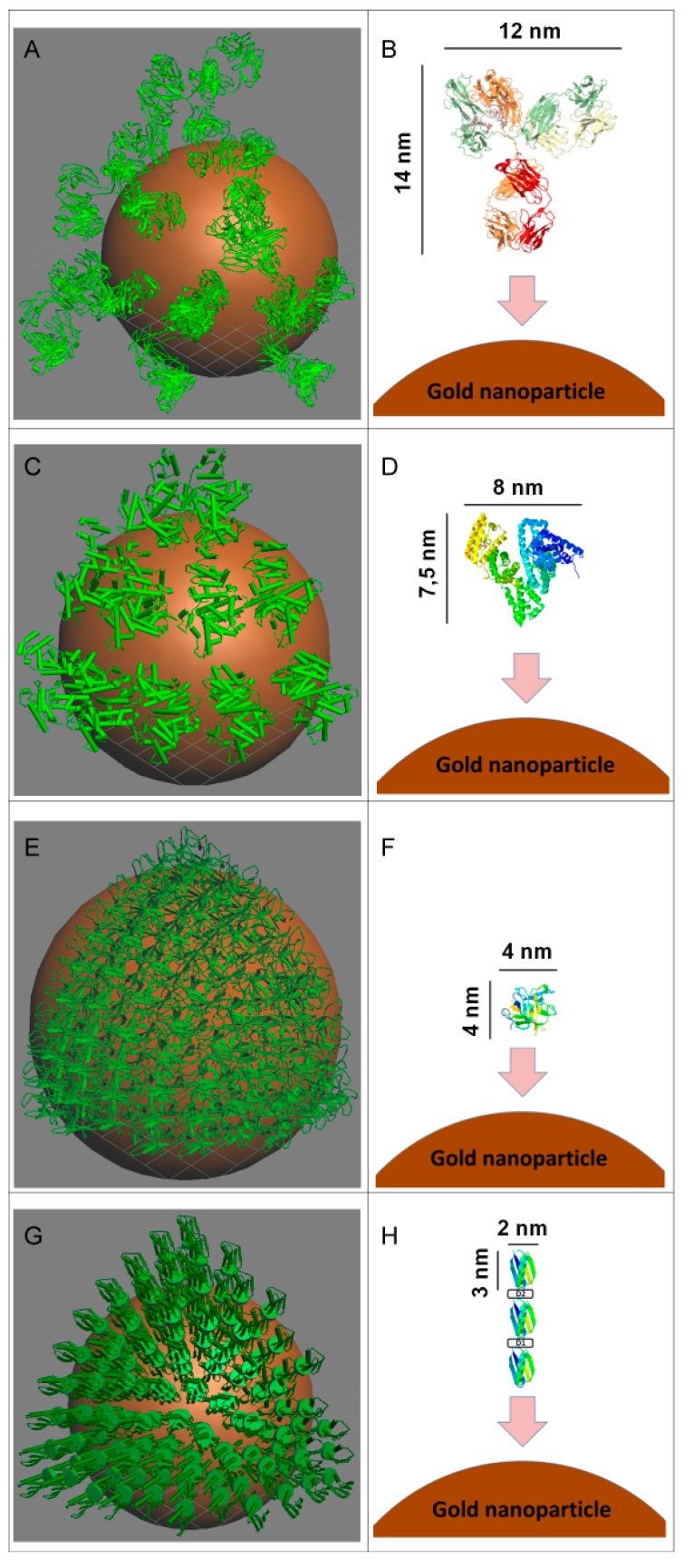
Images of the proteins used in the study and their conjugates with gold nanoparticles (ø 24 nm). (**A**,**B**) for immunoglobulin G (IgG); (**C**,**D**) for bovine serum albumin (BSA); (**E**,**F**) for Kunitz-type soybean trypsin inhibitor (STI); (**G**,**H**) for recombinant streptococcal protein G (protein G). Figures **B**, **D**, **F**, **H** show orientations of proteins on the nanoparticle surface for models given at figures **A**, **C**, **E**, **G**, respectively.

As an illustration of this hypothesis, we constructed three-dimensional models of these conjugates. A sphere 24 nm in diameter was created as an analog of the nanoparticle; protein models were placed on its surface. Figure 7 shows models of the colloid conjugates with coverage of 1/8th of the nanoparticle surface. The following numbers of protein molecules were placed on the nanoparticle surface: IgG-6; BSA-11; STI-60; and protein G-63. Thus, for the given density of surface coverage, the total number of protein molecules for the entire sphere was 48, 88, 480 and 504 molecules for IgG, BSA, STI, and protein G, respectively. These values are comparable to the data in Table 1. Thus, the experimentally determined amounts of adsorbed proteins for the saturation conditions could form a protein monolayer on the surface of the studied nanoparticle.

The fluorescence properties of proteins vary greatly depending on their internal and external environment. The particularity of the proposed approach consists in the fact that the fluorescence is measured at a particular wavelength in the same condition, namely in the presence of supernatant obtained during the conjugation. Thus, the same conditions are provided for all the compared preparations, and the recorded fluorescence reflects only the protein concentration, and does not reflect other factors. The calculations are based only on the comparison of the parameters obtained for the same protein that was added to the nanoparticles and is not associated with them. Therefore the differences between proteins in the content of fluorescent amino acids do not affect the correctness of the calculations. This feature is distinguished the proposed methodology exactly from other approaches, which involve fluorescent data obtained under different conditions to characterize processes of protein immobilization (e.g., values of fluorescence for protein in solution and for immobilized protein).

## 3. Materials and Methods

### 3.1. Reagents

Bovine serum albumin (BSA) and chloroauric acid were from Sigma-Aldrich (St. Louis, MO, USA). Kunitz-type soybean trypsin inhibitor (STI) was from MP Biomedicals (Santa Ana, CA, USA), protein G and human IgG were from Imtek (Moscow, Russia). The protein G used in the work is a recombinant mutant with a molecular mass of 26 kD, and consists of three IgG-binding fragments [24].

All salts were of analytical or reagent grade. Deionized water, with a resistance of 18.2 MV/cm at 25 °C, was obtained using a Simplicity system from Millipore (Billerica, MA, USA) and used to prepare all aqueous solutions.

### 3.2. Preparation of Gold Nanoparticles

Gold nanoparticles were synthesized according to the Frens method [38]. A chloroauric acid (5%) solution (0.2 mL) was added to water (97.5 mL), heated to boiling, and then 1% sodium citrate solution (1.5 mL) was added with stirring. The mixture was boiled for 25 min, cooled to room temperature, and stored at 4 °С. The pH of the solution was 5.4.

### 3.3. Transmission Electron Microscopy

Transmission electron microscopy images of the gold nanoparticles were obtained following an established procedure [39]. Gold nanoparticles were mounted on 300-mesh grids (SPI Supplies, West Chester, PA, USA), precoated with a support film of poly(vinyl formal) dissolved in chloroform. The images were obtained with a CX-100 electron microscope (CX-100, Jeol, Tokyo, Japan) at an accelerating voltage of 80 kV and magnification of 3,300,000. 

Negative films were scanned in gray scale mode on the scanner to obtain images with a resolution of 1200 dpi. The images are saved in tiff format and processed using Image Tool software (University of Texas Health Science Centre at San Antonio, San Antonio, TX, USA). The average particle size and size distribution were calculated.

### 3.4. Dynamic Light Scattering

Dynamic light scattering of gold nanoparticles was recorded with a Photocor spectrometer (Photocor Instruments, College Park, MD, USA) equipped with a helium–neon laser (632.8 nm). The hydrodynamic radius distribution of the gold nanoparticles was obtained at 25 °C by measuring the intensity of scattered light at an angle of 90°. The results of the measurements were processed with DynaLS version 2.5.2 (Alango, Tirat Carmel, Israel). Each experiment was repeated ten times.

### 3.5. Preparation of the Gold Nanoparticle-Protein Conjugates

Eight 2.0 mL tubes were filled with a solution of gold nanoparticles, and then centrifuged at 12,000× *g*. The supernatant was collected. The pellet of nanoparticle was agitated, and the volume of liquid in the tube was adjusted up to 0.2 mL using the supernatant. The supernatant was used to dilute a protein stock solution (100 mg/mL) to 1000, 500, 250, 125, 62.5, 31, 16, and 8 μg/mL. The protein solutions were freshly prepared before each experiment.

An aliquot (0.2 mL) of each diluted protein solutions was added to 0.2 mL of the gold nanoparticles solution remaining after centrifugation. The protein and gold nanoparticle mixtures were incubated for 1 h at room temperature and then centrifuged at 12,000× *g*. After the centrifugation aliquots (0.2 mL) of supernatant were taken and transferred to the microplate for fluorescence measurements.

### 3.6. Fluorescence Measurements

The fluorescence spectra of proteins were recorded on a microplate reader (Perkin Elmer En Spire 2300, Waltham, MA, USA). Spectra were measured using Nunc MaxiSorp white microplates (Roskilde, Denmark), with excitation and emission wavelengths of 280 nm and 290–500 nm, respectively (See Supporting Information 1).

### 3.7. Three-Dimensional Modeling the Gold Nanoparticle-Protein Conjugates

Models of the conjugates were constructed using 3DS Max 6 software (Autodesk, Inc., San Rafael, CA, USA). Three-dimensional models of proteins were taken from the RCSB PDB database. The PDB codes were as follows: IgG, 1IGY; BSA, 4F5S; STI, 1AVU; and protein G (IgG-binding fragment), 1PGB. The recombinant mutant of protein G consisting of three IgG-binding fragments, D1 and D2 intermediate fragments, was used. The proteins models were converted to VRML World File (*.wrl) format using DS Viewer Pro software (Afanche Technologies, Inc., Hinsdale, IL, USA).

## 4. Conclusions

A simple method was developed for the determination of the composition of protein conjugates based on the measurement of the fluorescence of tryptophan residues in the proteins. Using this method, conjugates between gold nanoparticles (diameter 24 nm) and four proteins (IgG, BSA, streptococcal protein G and STI) were studied. This method could be suitable for the analysis of protein conjugates with other nanoparticles.

## Supplementary Materials

Supplementary materials can be found at http://www.mdpi.com/1422-0067/16/01/0907/s1.
